# Quality of life in Italian patients with Multiple endocrine neoplasia type 1 (MEN 1): results of an extensive survey

**DOI:** 10.1186/s13023-020-01650-y

**Published:** 2021-01-06

**Authors:** Francesca Giusti, Federica Cioppi, Caterina Fossi, Francesca Marini, Laura Masi, Francesco Tonelli, Maria Luisa Brandi

**Affiliations:** Department of Experimental and Clinical Biomedical Sciences, University of Florence, Largo Palagi 1, 50139 Florence, Italy

**Keywords:** Multiple endocrine neoplasia type 1, Neuroendocrine tumors, Quality of life, LOT-R, IES-R, HADS, SF-36

## Abstract

**Background:**

MEN1 is a complex, rare, syndrome inherited in an autosomal dominant tract and characterized by the development of multiple neuroendocrine tumors, requiring lifelong surveillance and multiple medical and surgical therapies throughout the patient’s life. For all these reasons, a diagnosis of MEN1 can be a psychological shock for the patient, as well as his/her relatives, more so than the diagnosis of a single tumor. In the last two decades, clinicians have started to consider the emotional, psychological, relational, and social aspects of their patients’ lives. The data collected in the present analyses highlight the unique features of MEN1 syndrome, and aim to evaluate the Quality of Life in the patients and their relatives. In this study, a comprehensive survey of various aspects of Health-Related Quality of Life was performed in a large series of Italian MEN1 patients, by administering five of the most common targeted questionnaires.

**Results:**

The results of the study showed that our patients, despite having a complex multi-tumor syndrome, were moderately optimistic (50%), and this corresponds with a normal Quality of Life. This positive response is strictly correlated with the fact that the patients are cared for at a dedicated Referral Center, receiving personalized care and constant follow-up, which gives them reassurance regarding the high quality of management of the disorder.

**Conclusions:**

The possibility of having access to a clinical Referral Center for their complex rare disease, together with the support of a dedicated patient association, has been demonstrated to be the ideal model for the management of post-diagnosis shock, and contributes to the preservation of a good Health-Related Quality of Life for MEN1 patients.

## Background

Multiple Endocrine Neoplasia type 1 (MEN1) is a rare hereditary multiple tumor syndrome, typically characterized by the development of multiple tumors, mainly parathyroid adenomas, neuroendocrine tumors of the gastro-entero-pancreatic tract, and adenomas of the anterior pituitary gland [1]. The disease is caused by inactivating mutations of the *MEN1* tumor suppressor gene; inherited in a transmission is autosomal dominant tract with a full penetrance after 50 years of age [1]. The main tumors that characterize MEN1 syndrome are adenomas of the parathyroid glands, insulinomas, duodenal and pancreatic gastrinomas, and adenomas of the anterior pituitary (prolactinoma, adrenocorticotropin-secreting and/or somatotropin-secreting tumors or non-secreting adenoma). Contrary to their sporadic counterparts, these MEN1 tumors onset at an earlier age, present a higher frequency of relapse, occur often in multifocal form, and manifest more aggressive clinical features.

In MEN1 patients, a constellation of over 20 different endocrine and non-endocrine tumors present, including bronchial and thymic carcinoids, lipomas, and, in a lesser percentage, meningiomas, collagenomas, facial angiofibromas, and leiomyomas [1]. In general, numerous variable combinations of the different tumors and lesions can occur, even within the same family, and even in homozygous twins [1]. Conversely, the same symptoms can occur in patients with different mutations of the *MEN1* gene. This broad phenotypic variability in MEN1 patients cannot be explained by genetics, and there is no defined genotype–phenotype correlation that allows clinicians to predict the clinical course of the disease, starting from the specific mutation of the *MEN1* gene, and thereby tailor a personalized mutation-driven preventive/therapeutic approach [2].

The possibility that epigenetic factors play a role has been considered, but not extensively evaluated in the population described in the literature.

All clinically diagnosed MEN1 patients and *MEN1* gene mutation carriers must undergo a specific program of diagnostic surveillance, consisting in routine biochemical and radiological screenings for their entire lives, beginning with the recognition of their MEN1 clinical status, or with the identification of a *MEN1* gene mutation. The occurrence of multiple tumors, together with consequent surgery, accounts for a reduction of quality of life for MEN1 patients.

The diagnosis of a tumor is a trauma for patients and their families, mainly due to the uncertainty of its outcome. Psychologists believe that depression, anxiety, and fear are normal responses following the first medical diagnosis. Indeed, clinical symptoms, such as pain, nausea, appetite changes, insomnia, and/or tiredness, are often associated with this critical moment [3].

For years, Health-Related Quality of Life (HRQoL) remained only a marginal aspect of MEN1, with clinicians focusing only on solving diagnostic and therapeutic issues of the disease.

Only during the last two decades has the evaluation of Quality of Life (QoL) in MEN1 patients attracted the attention of the medical community as an important medical aspect to be considered in the management of patients with this complex syndrome.

In 2003, a Swedish study demonstrated, for the first time, the psychosocial distress following the diagnosis of MEN1 in 29 MEN1 patients, with a higher degree of depression affecting patients categorized as having a high burden of disease and treatment [3]. Later, in 2007, a follow-up study on the same patients investigated how they lived with the disease, and showed that patients did learn to live with their disease, the majority declaring that they felt healthy, despite physical symptoms and treatments [4]. These data demonstrate that constant and personalized care and follow-up can significantly improve psychological status and perception of the disease.

An increase of our knowledge about emotional, psychological, relational, and social aspects of MEN1-related QoL in affected and/or diagnosed patients will help us provide them with the best care, reducing the negative influence of a pessimistic approach to the management of the disease.

The present study aimed to perform a comprehensive survey about the individual perception of disease and HRQoL, in terms of both physical status and psychological, emotional, social, and economic impacts. For the first time, we have analyzed HRQoL in a large series of Italian MEN1 patients by using the simultaneous administration of five of the most commonly applied clinical questionnaires.

## Results

### Population main clinical characteristics

Seventy-six patients participated in the study and completed all five questionnaires [52 (68.42%) females and 24 (31.58%) males] (Table 1). The mean age was 47.12 ± 14.13 years (range 19–79) at the time of inclusion in this study.

**Table 1:** Socio-demographic characteristics of our MEN1 patients

Characteristic	No. of cases	%
*Gender*		
Females	52	68.42
Males	24	31.58
*Social status*		
Single	24	31.58
Married/cohabitant	38	50
Divorced/separated	7	9.21
Widow/widower	2	2.63
Girlfriend/boyfriend	5	6.58
*Children*		
Yes	43	56.58
No	33	43.42
*Education*		
Primary school	18	23.68
High school	38	50
College or university	20	26.32
*Employment*		
Employee	37	48.69
Freelance	10	13.15
Retired	8	10.54
Housewife	3	3.95
Under education	2	2.63
Unemployed	10	13.15
Not able to work due to illness	6	7.89
*Smoke*		
Yes	11	14.47
No	65	85.53
*Do you exercise for more than 30 min at least 3 times a week?*		
Yes	43	56.58
No	33	43.42
*Due to the MEN1, has your lifestyle changed?*		
Yes	23	30.26
No	53	69.74
*Has MEN1 affected your work negatively?*		
Yes	27	35.52
No	49	64.48
*Has MEN1 limited your earnings?*		
Yes	23	30.26
No	53	69.74
*How far is your reference center from your home?*		
Mean 324 km	n.a	n.a
*How long does it take you to reach your referral center from your home?*		
Mean 310 min	n.a	n.a
*Do you receive home care for your illness?*		
No	76	100

*n.a.* non available

The analysis of family histories allowed us to identify 67 (88.16%) familial cases belonging to 36 different pedigrees, and 9 (11.84%) sporadic cases. A *MEN1* mutation was identified in 68/76 (89.47%) patients; of them, 61 were familial cases belonging to 32 pedigrees, and 7 were sporadic cases. Conversely, no mutations in the coding region and splicing sites of the *MEN1* gene were detected in 8 patients (10.53%); of them, 6 were familial cases belonging to 4 families and 2 were sporadic cases.

The mean age at the first clinical manifestation was 29.20 ± 12.49 years (range 15–59). The three most prevalent lesions typical of MEN1 in our series were 71 primary hyperparathyroidism (PHPT) (93.42%), 50 gastro-entero-pancreatic neuroendocrine tumors (GEP-NETs) (65.78%), and 48 pituitary tumors (63.15%). The most frequent clinical phenotype resulted to be combination PHPT/GEP-NETs/pituitary tumors (35 cases; 46.05%), followed by PHPT/GEP-NETs (13 cases; 17.10%), PHPT/pituitary tumors (12 cases; 15.79%), and pituitary tumors/GEP-NETs (3 cases; 3.94%). One relative included in the database remained disease-free for the duration of the study; namely, no abnormalities in biochemical tests or evidence of disease by instrumental examinations were detected. This subject, diagnosed only by *MEN1* genetic testing, was 30 years of age at the time of this study.

The total number of surgeries in a single patient varied between 0 and 4, with a mean of 1.3: sixteen patients underwent no surgery for MEN1 related diseases, 30 patients underwent at least 1 surgery, 20 patients had two interventions, 9 patients three interventions, and 1 patient underwent four interventions. The most common surgeries were in the parathyroids (64.47%), followed by the pancreas (35.52%), lipomas (22.36%), bronchial carcinoids (10.52%), thymic carcinoids (2.63%) and pituitary tumors (2.63%). In detail, parathyroid surgeries consisted of 21 total parathyroidectomies, all with auto-transplantation of healthy parathyroid tissue in the forearm (of them 2 were second surgeries for recurrence, after a previous partial parathyroidectomy), 20 subtotal parathyroidectomies (with removal of 3 glands and part of the forth one) and 10 partial parathyroidectomies (with only the adenomatous gland being removed). Pancreatic surgeries consisted of 9 pancreato-duodenectomies, one pancreato-duodenectomy associated with single tumor enucleation in the remaining pancreas, 12 body-tail pancreatic resections, 2 body-tail pancreatic resections associated with single tumor enucleation, and 3 single tumor enucleations. No total pancreatoctomy was performed in our cohort of patients. None of our patients required insulin therapy after pancreatic surgery. Pituitary surgeries were all performed by the trans-sphenoidal approach; none of our patients underwent pituitary radiotherapy.

The number of drugs taken by the study population to treat the manifestation of MEN1 varied from one to six. In detail, medical treatments consisted of: vitamin D in 57 patients, dopamine agonists in 26, calcium supplementation in 25, somatostatin analogues in 20, pancreatic enzymes in 17, gastric protectors in 12, oral antidiabetic agents in 8 (5 after pancreatic surgeries, one in presence of a pancreatic NET medically treated with somatostatin analogue, one for diabetes caused by chronic corticosteroid therapy for psoriatic arthritis, and one to treat second-grade obesity), calcimimetics in 7, anti-inflammatories in 5, analgesics in 3, testosterone in one, and potassium citrate in one.

Twenty patients, 12 females (60%) and 8 males (40%), did not accepted to participate in this study (mean age 51.11 ± 12.93 years; range 27–72). Their mean age at the first MEN1 clinical manifestation was 36.25 ± 12.57 years (range 15–65). These included 18 familial cases (90%) belonging to 13 different pedigrees, and 2 sporadic cases (10%). A *MEN1* mutation was identified in 19/20 (95%) patients. No mutation in the coding region and splicing sites of the MEN1 gene was detected in one patient (5%). They accounted for 19 PHPT (95%), 12 GEP-NETs (60%), and 11 pituitary tumors (55%), with the most frequent clinical phenotype resulting to be combination PHPT/GEP-NETs/pituitary tumors (5 cases; 25%), followed by PHPT/GEP-NETs (5 cases; 25%), and PHPT/pituitary tumors (3 cases; 15%). The total number of surgeries in a single patient varied between 0 and 4, with a mean of 1.25; five patients underwent no surgery for MEN1 related diseases, 8 patients underwent at least 1 surgery, 5 patients had two surgeries, 1 patient three surgeries, and 1 patient underwent four surgeries. The most common surgeries were of the parathyroids (55%), followed by pancreas (25%), lipomas (20%), and pituitary tumors (20%). The number of drugs taken to treat the manifestation of MEN1 varied from one to five.

### Socio-demographic questionnaire

The patients in this sample were mostly married or cohabitant (50%), followed by single (31.58%), divorced or separated (9.21%), girlfriend or boyfriend (6.58%), and widow or widower (2.63%).

50% had a high school education, followed by college or university (26.32), and primary school only (23.68%). Most patients worked as employees (48.69%), followed by freelance (13.15%), unemployed (13.5%), retired (10.54%), not able to work due to illness (7.89%), housewife (3.95%), and still in school (2.63%).

Eighty-six of the patients did not smoke; 56.58% were physically active.

In 69.74% of cases, MEN1 did not change the patient’s lifestyle.

In 64.48% of cases, MEN1 had no negative effects on work.

In 69.74% of cases, MEN1 did not limit earnings.

The Center of Reference is an average of 324 km from the home of the patients included in the study, and they take an average of 310 min to reach it.

None of the patients received home care for MEN1.

The socio-demographic data of our study population are summarized in Table 1.

### Life Orientation Test-Revised (LOT-R)

**Fig. 1:**
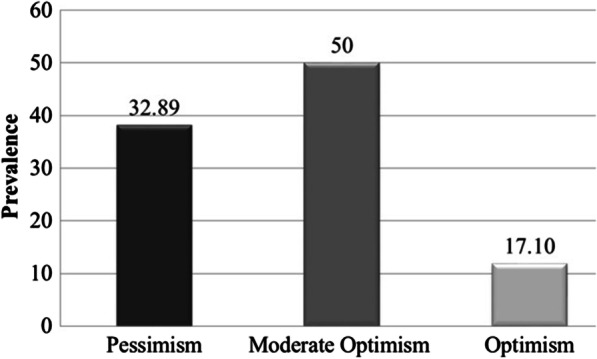
Prevalence of pessimism, assessed by the LOT-R questionnaire

The prevalence of pessimism was of 32.89% (25 patients), moderate optimism 50% (38 patients) and high optimism 17.10% (13 patients) (Fig. 1).

The average of total LOT-R score was 14.93 ± 4.12 (range 0–24). The score for pessimism was 10.24 ± 2.06 (range 0–13), for moderate optimism 15.89 ± 1.39 (range 14–18), and for high optimism 21.07 ± 1.49 (range 19–24).

The most frequently measured value was 15, the maximum detected value was 23, and the minimum value was 6.

### Impact of Event Scale-Revised (IES-R)

**Fig. 2:**
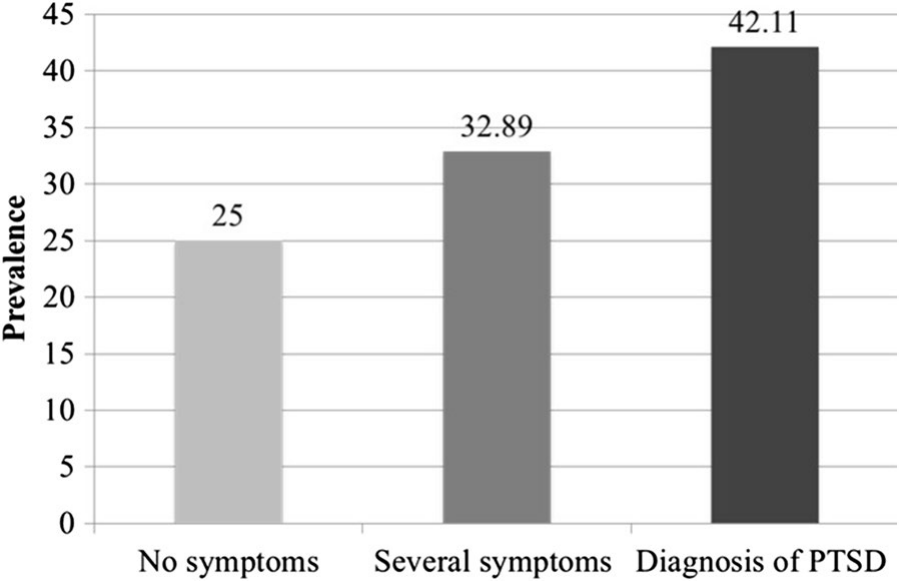
Prevalence symptoms of post-traumatic stress disease, assessed by IES-R questionnaire

The prevalence of little or no symptoms of post-traumatic stress disorder (PTSD) was 25% (19 patients); several symptoms of PTSD 32.89% (25 patients); patients with diagnosis of PTSD 42.11% (32 patients) (Fig. 2).

The average of total IES-R score was 29.52 ± 19.03 (range 1–88). The score for little or no symptoms was 6.84 ± 4.24 (range 1–11). The score for several symptoms was 23.00 ± 6.73 (range 12–32), and that for patients with diagnosis of PTSD 48.09 ± 11.19 (value ≥ 33).

The intrusiveness dimension score was 1.51 ± 0.36, avoidance dimension score 1.30 ± 0.35, and hyperarousal dimension score 1.23 ± 0.32.

### Hospital Anxiety and Depression Scale (HADS)

**Fig. 3:**
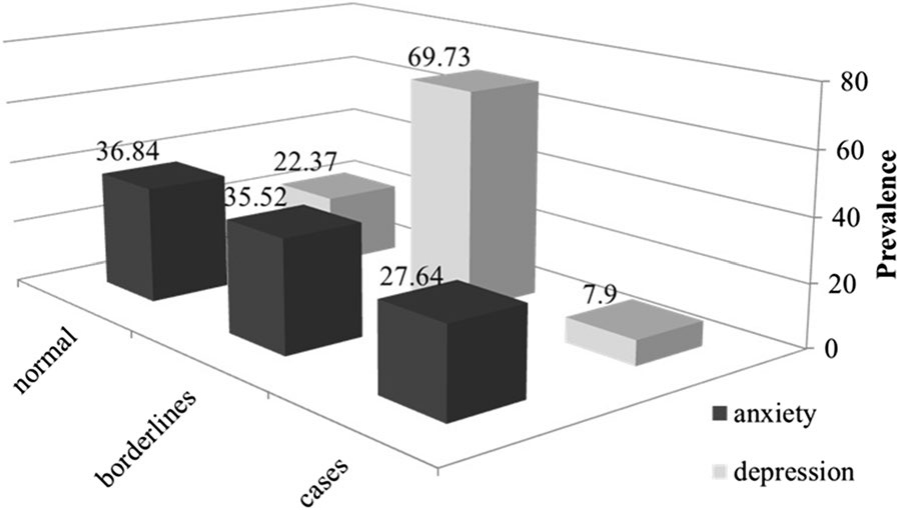
Prevalence anxiety and depression, assessed by HADS questionnaire

The prevalence of anxiety indicated as normal cases was 36.84% (28 patients), borderline cases 35.52% (27 patients), and major cases 27.64% (21 patients) (Fig. 3).

The average of total anxiety score was 8.78 ± 2.60 (range 0–21). The average score for normal cases was 6.25 ± 1.00 (range 0–7), for borderline cases 8.77 ± 0.84 (range 8–10), and for major cases 12.19 ± 1.40 (value ≥ 11).

The most frequent measured value was 7, the maximum detected value was 15, and the minimum value was 5.

The prevalence of depression indicated as normal cases was 22.37% (17 patients), borderline cases were 69.73% (53 patients), and major cases 7.90% (6 patients) (Fig. 3).

The average of total depression score was 8.68 ± 1.62 (range 0–21). The average score for normal cases was 6.35 ± 0.86 (range 0–7), for borderline cases 9.09 ± 0.68 (range 8–10), and for major cases 11.67 ± 1.21 (value ≥ 11).

The most frequently measured value was 9, the maximum value detected was 14, and the minimum value was 5.

A statistically significant increase in anxiety (p < 0.05) with increasing age was found, particularly among young adults (18–44 years) and adults (45–60 years).

### Medical Outcomes Study 36-item Short-Form Health Survey (SF-36)

**Fig. 4:**
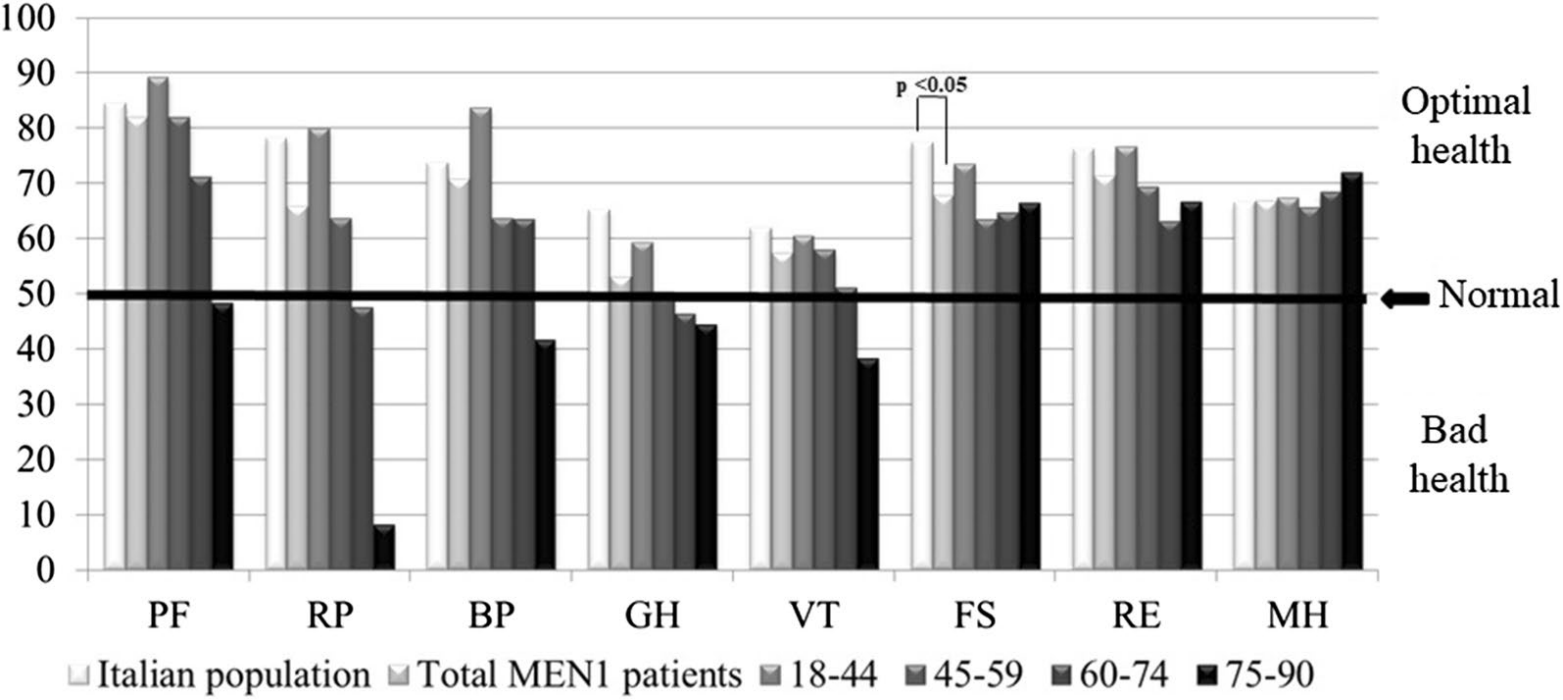
Averages of scores attributed to quality of life variables, assessed by SF-36 questionnaire, stratified by age

The averages of the 8 dimensions were: physical functioning 82.01 ± 21.39 (range 25–100); physical role 65.78 ± 40.58 (range 0–100 range); body pain 70.68 ± 26.98 (range 10–100); general health 53.01 ± 22.53 (0–97); vitality 57.30 ± 20.44 (range 10–100); social functioning 67.75 ± 24.40 (range 25–100); emotional role 71.27 ± 38.44 (range 0–100) and mental health 66.88 ± 17.14 (range 32–100) (Table 2). The values ​​of the 8 dimensions were all satisfactory and above the average of the Italian population (Table 2). The physical functioning dimension had the highest average score, while the general health dimension had the lowest. Figure 4 shows the results of SF-36 stratified by different age groups.

**Table 2:** Scores of SF-36 variables in the Italian population and in MEN1 patients

	n	SF-36							
		PF	RP	BP	GH	VT	SF	RE	MH
*Italian population*	2031								
Mean		84.5	78.2	73.7	65.2	61.9	77.4^a^	76.2	66.6
SD ±		23.2	35.9	27.7	22.2	20.7	23.3	37.3	20.9
*MEN1 patients*	76								
Mean		82.01	65.78	70.68	53.01	57.30	67.75^a^	71.27	66.88
SD ±		21.39	40.58	26.98	22.53	20.44	24.40	38.44	17.14
range		25–100	0–100	10–100	0–97	10–100	25–100	0–100	32–100

*PF* Physical functioning, *RP* Role limitations due to physical health problems, *BP* Bodily pain, *GH* General health perceptions, *VT* Vitality, *SF* Social functioning, *RE* Role limitations due to emotional problems, *MH* General mental health

^a^p < 0.05

### Overall analyses

Tables 3 and 4 illustrate the results of the five questionnaires stratified by gender and age group, respectively.

**Table 3:** Scores of HADS, IES-R, LOT-R and SF36 questionnaires in MEN1 patients, stratified by gender

	n	Age	Surgeries	HADS-A	HADS -D	LOT-R	IES-R	SF-36							
								PF	RP	BP	GH	VT	SF	RE	MH
*Males*	24														
Mean		47.04	1.33	8.5	8.66	14.58	25.5	89.85^a^	67.70	72.79	59.66	60.58	73.83	71.83	64.75
SD ±		13.49	0.91	2.65	1.65	3.83	19.86	14.81	38.64	26.84	18.85	18.02	24.69	37.77	17.14
*Females*	52														
Mean		47.49	1.32	8.92	8.69	15.09	31.38	78.38^a^	64.90	69.71	49.94	55.78	64.94	71.01	67.86
SD ±		14.48	1.02	2.59	1.62	4.28	18.53	23.05	41.79	27.25	23.57	21.46	23.98	39.11	17.22

*HADS* Hospital Anxiety and Depression Scale, *HADS-A* scores for anxiety, *HADS-D* scores for depression, *LOT-R* Life Orientation Test-Revised, *IES-R* Impact of Event Scale-Revised, *SF-36* Variables of Study 36-item Short-Form Health Survey, *PF* Physical functioning, *RP* Role limitations due to physical health problems, *BP* Bodily pain, *GH* General health perceptions, *VT* Vitality, *SF* Social functioning, *RE* Role limitations due to emotional problems, *MH* General mental health

^a^p < 0.05

**Table 4:** Scores of HADS, IES-R, LOT-R and SF36 in MEN1 patients, stratified by age

	n	Age	HADS-A	HADS -D	LOT-R	IES-R	SF-36							
							PF	RP	BP	GH	VT	SF	RE	MH
*18–44 years*	30													
Mean		32.96	8.1^a^	8.66	14.06	25.63	89.16 ^a^	80^a^	83.66 ^a^	59.33	60.53	73.56	76.56	67.3
SD ±		7.19	2.09	1.80	3.78	18.60	17.26	30.37	20.46	17.86	20.10	23.04	36.33	17.49
*45–59 years*	33													
Mean		51.78	9.63^a^	8.66	14.42	31.09	81.84	63.63	63.72^a^	50.06	57.96	63.51	69.36	65.57
SD ±		4.18	2.77	1.63	3.04	18.12	19.87	42.43	28.22	27.25	21.09	25.81	39.47	18.55
*60–74 years*	10													
Mean		65.5	8.7	8.7	15.1	39.4	71.2^a^	47.5^a^	63.4^a^	46.4	51.1	64.7	63.1	68.4
SD ±		3.02	2.40	1.41	3.31	20.89	26.89	46.32	27.22	17.21	16.25	26.09	39.91	12.85
*75–90 years*	3													
Mean		77	6.66	9	16.66	18.33	48.33^a^	8.33^a^	41.66^a^	44.33	38.33	66.33	66.66	72
SD ±		2	4.04	0	3.05	20.03	14.43	14.43	17.03	10.69	24.66	7.50	57.73	16

*HADS* Hospital Anxiety and Depression Scale, *HADS-A* scores for anxiety, *HADS-D* scores for depression, *LOT-R* Life Orientation Test-Revised, *IES-R* Impact of Event Scale-Revised, *SF-36* Variables of Study 36-item Short-Form Health Survey, *PF* Physical functioning, *RP* Role limitations due to physical health problems, *BP* Bodily pain, *GH* General health perceptions, *VT* Vitality, *SF* Social functioning, *RE* Role limitations due to emotional problems, *MH* General mental health

^a^p < 0.05

Table 5 shows the results of the five questionnaires according to the social aspects of the patients’ lives (i.e. marital status, education, and employment status).

**Table 5:** Scores of HADS, IES-R, LOT-R and SF36 in MEN1 patients, stratified by marital, educational and employment status

	n	Age	HADS-A	HADS -D	LOT-R	IES-R	SF-36							
							PF	RP	BP	GH	VT	SF	RE	MH
*Married/cohabitant + Girlfriend/boyfriend*	43													
Mean		50.60	8.93	8.74	14.34	30.48	80.76	68.60	67.79	50.76	53.81	67	74.93	65.20
SD ±		12.05	2.97	1.63	3.37	18.64	22.63	39.37	27.83	23.21	20.75	24.62	37.95	16.94
*Single*	33													
Mean		42.57	8.60	8.60	14.60	28.27	83.63	62.12	74.45	55.93	61.84	68.72	66.51	69.06
SD ±		15.48	2.04	1.63	3.40	19.74	19.88	42.44	25.75	21.60	19.41	24.45	39.14	17.42
*Employee/freelance*	47													
Mean		45.04	8.87	8.59	14.87	26.85	88.68^a^	75.5 ^a^	74.89	57.0^a^	60.51	68.44	74.93	68.17
SD ±		10.56	2.49	1.55	3.23	17.49	15.46	35.15	26.96	23.33	22.10	25.21	37.19	19.31
*No working*	29													
Mean		50.48	8.65	8.82	13.79	33.86	71.20^a^	50^a^	63.86	46.51^a^	52.10	66.62	65.34	64.7
SD ±		18.24	2.80	1.75	3.53	20.88	25.24	44.32	26.03	19.86	16.48	23.43	40.34	12.94
*Primary school*	18													
Mean		47.88	9.5	7.88^a^	14.16	31	72^a^	51.38	65.61	47.88	59.77	65.83	67.88	66.11
SD ±		11.08	3.22	1.56	2.99	20.28	25.23	42.41	27.97	25.27	20.85	24.17	33.39	19.16
*High school*	38													
Mean		47.84	8.55	8.92^a^	15	31.15	86.28^a^	70.39	70.89	54.34	54.31	66.92	68.31	64.28
SD ±		15.36	2.46	1.73	3.27	20.53	18.75	38.05	27.37	20.52	21.71	24.16	41.01	17.87
*College or university*	20													
Mean		45.05	8.6	8.95^a^	13.7	25.1	82.9	70	74.85	55.1	60.75	71.05	79.95	72.5
SD ±		14.59	2.23	1.27	3.84	14.57	20.47	42.61	25.90	24.07	17.49	25.97	38.11	12.74

*HADS* Hospital Anxiety and Depression Scale, *HADS-A* scores for anxiety, *HADS-D* scores for depression, *LOT-R* Life Orientation Test-Revised, *IES-R* Impact of Event Scale-Revised, *SF-36* Variables of Study 36-item Short-Form Health Survey, *PF* Physical functioning, *RP* Role limitations due to physical health problems, *BP* Bodily pain, *GH* General health perceptions, *VT* Vitality, *SF* Social functioning, *RE* Role limitations due to emotional problems, *MH* General mental health

^a^p < 0.05

Tables 6 and 7 summarize the results of the five questionnaires in relation to MEN1 therapies (respectively, the number of surgeries and the number of administrated drugs for the treatment of MEN1 manifestations).

**Table 6:** Scores of HADS, IES-R, LOT-R and SF36 in MEN1 patients, stratified by number of surgeries

	n	Age	HADS-A	HADS -D	LOT-R	IES-R	SF-36							
							PF	RP	BP	GH	VT	SF	RE	MH
*0 surgeries*	16													
Mean		38.6	9.43	9.06	14.18	29.18	87.5	75^**a**^	85.43^**a**^	58.81^**a**^	56.87	72.5^**a**^	66.62	66.43
SD ±		15.92	2.25	2.23	4.41	20.86	19.52	37.63	22.51	20.80	20.23	24.24	47.14	16.34
*1 surgeries*	30													
Mean		49.43	8.1	8.3	15.23	25.03	80.46	63.33	70.66	53.4	58.9	69.8	74.3	69.06
SD ±		14.82	2.26	1.55	3.15	19.15	21.40	41.38	26.56	19.34	18.29	23.13	36.87	16.27
*2 surgeries*	20													
Mean		49.55	9.3	8.75	15.15	35.4	82.3	70	64.45^**a**^	54.2	54.25	68.5	71.5	62.8
SD ±		11.83	3.02	1.33	4.33	18.56	23.14	41.03	27.65	25.37	23.07	22.29	36.40	15.79
*3 surgeries*	9													
Mean		49.44	9.11	9.22	14	31.77	75.33	44.44^**a**^	55.11^**a**^	38.55^**a**^	55.33	47.22^**a**^	65.77	65.77
SD ±		8.98	3.05	1.09	5.8	16.20	22.41	41.03	24.26	27.48	21.38	26.35	37.40	22.45
*4 surgeries*	1													
		53	6	8	22	32	95	100	100	55	95	100	100	100

*HADS* Hospital Anxiety and Depression Scale, *HADS-A* scores for anxiety, *HADS-D* scores for depression, *LOT-R* Life Orientation Test-Revised, *IES-R* Impact of Event Scale-Revised, *SF-36* Variables of Study 36-item Short-Form Health Survey, *PF* Physical functioning, *RP* Role limitations due to physical health problems, *BP* Bodily pain, *GH* General health perceptions, *VT* Vitality, *SF* Social functioning, *RE* Role limitations due to emotional problems, *MH* General mental health

^a^p < 0.05

**Table 7:** Scores of HADS, IES-R, LOT-R and SF36 in MEN1 patients, stratified by number of drugs taken for MEN1 treatment

Drugs	n	Age	HADS-A	HADS -D	LOT-R	IES-R	SF36							
							PF	RP	BP	GH	VT	SF	RE	MH
*0–2* (1.43 ± 0.50)	41													
Mean		44.60	8.82	8.63	15.36 ^a^	28.53	88.02 ^a^	75.60 ^a^	77.75 ^a^	57.51^a^	59.53	72.97 ^a^	72.26	65.48
SD ±		14.08	2.64	1.66	3.41	20.97	18.79	36.86	23.69	20.38	19.48	22.94	40.11	17.76
*3–4* (3.34 ± 0.48)	29													
Mean		50.24	8.55	8.62	13.51 ^a^	29.31	77.65 ^a^	61.20	65.65	51.44	56.86	65.79	74.55	69.17
SD ±		10.22	2.30	1.63	3.03	16.85	20.55	41.50	28.45	23.70	22.03	24.72	36.41	16.29
*5–6* (5.16 ± 0.37)	6													
Mean		49.16	9.66	9.33	12.83	37.33	62 ^a^	20.83 ^a^	46.66 ^a^	29.83^a^	44.16	41.5 ^a^	48.66	65.33
SD ±		15.51	3.82	1.36	3.25	15.60342	28.07	29.22	25.37	17.89	16.25	15.02	34.41	18.70

*HADS* Hospital Anxiety and Depression Scale, *HADS-A* scores for anxiety, *HADS-D* scores for depression, *LOT-R* Life Orientation Test-Revised, *IES-R* Impact of Event Scale-Revised, *SF-36* Variables of Study 36-item Short-Form Health Survey, *PF* Physical functioning, *RP* Role limitations due to physical health problems, *BP* Bodily pain, *GH* General health perceptions, *VT* Vitality, *SF* Social functioning, *RE* Role limitations due to emotional problems, *MH* General mental health

^a^p < 0.05

Table 8 summarizes the results of the five questionnaires in relation with the distance of the patient from the Reference Center.

**Table 8:** Distance of the patient from the Reference Center

	n	Age	HADS-A	HADS -D	LOT-R	IES-R	SF36							
							PF	RP	BP	GH	VT	SF	RE	MH
*0–200 km* (85.63 ± 60.19 km) (3.19 ± 10.74 h)	32													
Mean		45.63	9.16	9.16 ^a^	13.96	28.76	84.46	66.66	73.63	51.1	58.46 ^a^	68.6 ^a^	70.76 ^a^	66.3
SD ±		14.90	2.62	1.46	3.53	14.98	17.97	41.69	26.37	23.63	20.30	26	37.99	14.92
*201–400 km* (335.17 ± 56.92 km) (3.68 ± 1.19 h)	29													
Mean		47.96	8.34	8.44	14.62	24.41	81.82	72.41	75.75	60.20	60.72	72.62	80.31	71.93
SD ±		15.07	2.303	1.45	3.42	18.58	23.13	38.58	24.41	19.35	18.47	21.65	31.62	15.89
*401–600 km* (491.66 ± 58.45 km) (6.83 ± 0.75 h)	6													
Mean		46.50	8.83	6.66 ^a^	14.16	29.66	81.5	45.83	55.16	46.66	40 ^a^	45.83 ^a^	22.16 ^a^	56
SD ±		10.50	2.78	1.75	2.13	13.39	21.94	36.79	25.95	21.14	17.60	18.81	40.35	23.46
*601–1400 km* (972.22 ± 216.66 km) (15.22 ± / 8.58 h)	9													
Mean		49	8.11	9.11	15.77	42.22	79.11	63.88	61.44	48.33	59.44	70.55	81.33	65.77
SD ±		13.47	2.89	1.83	3.70	27.65	25.06	45.26	34.24	23.89	24.55	23.29	33.86	18.45

*HADS* Hospital Anxiety and Depression Scale, *HADS-A* scores for anxiety, *HADS-D* scores for depression, *LOT-R* Life Orientation Test-Revised, *IES-R* Impact of Event Scale-Revised, *SF-36* Variables of Study 36-item Short-Form Health Survey, *PF* Physical functioning, *RP* Role limitations due to physical health problems, *BP* Bodily pain, *GH* General health perceptions, *VT* Vitality, *SF* Social functioning, *RE* Role limitations due to emotional problems, *MH* General mental health

^a^p < 0.05

## Discussion

For decades, medical approaches to diseases, including tumors, have focused only on the management and treatment of clinical and pathological manifestations directly related to the specific disorder. Modern medicine has recognized that consideration of the patient’s QoL is of fundamental importance in determining the patient’s care, and it is now common to assess the emotional, relational, and psychological status of the patient as a central dimension in clinical practice, research, and health policy. At the origin of these developments, there are scientific, clinical, and ethical reasons. In particular, the patient's experience of illness must be integrated with the treatment, and the impact of mental discomfort, its consequences, and the effects of the treatment must be evaluated.

Although there is still no complete agreement on the definition and measurement of QoL, in recent years there has been an increasing recognition of its subjectivity, and the concept of QoL as a multidimensional construct. The World Health Organization defines QoL as "an individual's perception of their position in life in the context of the culture and value systems in which they live and in relation to their goals, expectations, standards and concerns.” [5].

Some studies have investigated the HRQoL in MEN1 patients by using questionnaires commonly used in other chronic or tumoral diseases, given that there are no dedicated questionnaires, and they have obtained interesting preliminary results.

Berglund et al. analyzed HRQoL in 29 out of 36 recruited Swedish MEN1 patients, through the administration of four questionnaires: IES and LOT in their non-revised versions, HADS, and SF-36. The questionnaires were administered twice: first, during a hospital visit, and second, six months later at home [3]. The scores obtained showed that SF-36-measured perception on their own general health and social outcomes were more negative in MEN1 patients than in the normal population. About 70% of MEN1 patients resulted to be pessimistic about their uncertain future, with fear of what might happen to them, their children, and other relatives. This pessimism may also be related to uncertainty about disease progression and how this could have a negative impact on their daily activities and their ability to maintain their current work situation. The authors showed that patients who presented more severe diseases and underwent extensive treatment may need support after discharge from the hospital for their psychosocial suffering. Strømsvik et al. [4] performed a follow-up study on the same 29 selected patients as Berlung et al. to evaluate how they live with the disease, through an interview conducted according to Griffin's 1986 guide, carried out by two psychologists, which focused on: quality of life, ability to influence and control own life, interpersonal relationships, and ability to achieve personal goals. The study showed that most of the participants tried to adapt to their new medical situation by changing their lifestyles and focusing on nutrition and physical activity. They signaled a shift in priorities after the development of MEN1 by learning about their personal risk. Changing values ​​helped patients manage the situation. Surprisingly, a majority of them described themselves as healthy, despite the severity of the disease, multiple surgeries, drug treatments, and physical and psychological symptoms. Participants reported that interpersonal relationships with family and friends were one of the most important aspects of their lives. Furthermore, a majority of participants indicated total satisfaction with being in a clinical surveillance and follow-up program, under the supervision of a multi-disciplinary group of specialists, as this allows immediate start of therapy at the time of tumor development, with a greater possibility of being treated definitively. As for work situations and environments, the test results highlighted the patient's sense of control, normally about working life, and only a small sense of fear about professional limitations related to the disease. Recently, Peipert et al. selected a group of MEN1 affected adult US citizens (from an initial population of 207 MEN1 patients), analyzing their HRQoL compared to other chronic diseases, through PROMIS (Patient-Reported Outcomes Measurement Information System), an initiative that aimed to develop novel ways to measure patient-reported symptoms, such as pain and fatigue, and aspects of HRQoL across a wide variety of chronic diseases and conditions. As PROMIS measures are disease agnostic, they can be “cross-cutting” in a meaningful way.

The authors showed that perception of HRQoL was worse, across PROMIS 29-item profile measure domains, and levels of anxiety, depression and fatigue were all higher in adults with MEN1 compared to both the general population and individuals affected by many other chronic conditions [6]. In 2018, Van Leeuwaarde et al. submitted a questionnaire to a total of 285 patients, 227 of whom (80%) were eligible as MEN1 patients. The questionnaire consisted of eight items adapted from the Cancer Worry Scale. This is an instrument to detect high levels of fear in patients with cancer. They reported that MEN1 patients with high fear of disease occurrence (FDO) had lower scores on the SF-36 scale, indicating a lower HRQoL. The study also showed that an increase in the number of MEN1 clinical manifestations was directly correlated to higher FDO scores [7].

In the present study, we analyzed the responses of 76 MEN1 patients to five different HRQoL questionnaires: a socio-demographic questionnaire, revised versions of LOT and IES, HADS, and SF-36. Data from the socio-demographic questionnaire indicated that, despite the disease, a majority of patients have been able to shift their priorities, managing the situation well, and making interpersonal relationships with family and friends one of the most important aspects of their lives. When asked if the MEN1 diagnosis had changed their lifestyle, negatively affected their work, and/or limited their earnings, about 60% of patients answered “no” to the questions. Our patients did not try to adapt to their new situation by changing their lifestyles, but maintained them, meanwhile focusing on physical activity and good habits, such as not smoking. Indeed, only 14.47% of patients smoke, while 56.58% practice physical activity for more than 30 min at least 3 times a week. We concluded that most patients consider themselves "healthy, despite everything", and maintain good control of their work lives as well.

The LOT-R test also showed that only 32.89% of the surveyed patients were pessimistic, a result clearly different from that obtained by the Berglund study [3]. Differently from our study, they used the LOT original version, a non-revised version of the test, which has been criticized by scientists for various aspects, such as the fact that it is not able to cover the scope of future expectations in as much detail as mentioned in its core theoretical base. However, despite the “positive” results of these two tests, the IES-R and HADS tests showed a percentage of cases reporting a worsening of their psychological health, which must be taken into account in tailoring patient management.

The IES-R test revealed the presence of symptoms of post-traumatic stress in 75% of our MEN1 patients: the most common symptom was intrusiveness, followed by avoidance and hyperarousal. Results of this test are a confirmation that a diagnosis of MEN1 is a shock, which causes an immediate psychological traumatic response in the patient. The high percentage of individuals showing signs of post-traumatic stress in our MEN1 cases is a strong indication that, after the communication of the MEN1 diagnosis, these patients have to be monitored over time, to prevent the development of depression or other long-term psychological disorders. The HADS test showed that the cases of major anxiety and depression were 27.64% and 7.90%, respectively. Given these relatively high percentages of patients at risk of developing anxiety and depression, it is probable that, along with these "pure" disorders, there is a mixed anxiety-depressive disorder. Our results confirmed that MEN1 patients should be followed up over time regarding their psychological status, and they should be given specific assessments for anxiety and/or depression evaluation. These data confirm those of Peipert [6], in which the scores of anxiety, depression and fatigue were statistically higher in MEN1 patients compared to other chronic conditions examined.

The SF-36 questionnaire showed that our MEN1 series was principally composed by people who have minimal difficulties in working or performing other daily activities due to emotional or physical problems, have good physical health, and believe that it is similar to that of their peers. They perform all types of activities, including the most demanding, without particular physical or emotional difficulties, since their perceived pain is not strong enough to limit performance, and their mood is positive. The average scores of SF-36 variables in the MEN1 patients were above the value of 50, which indicates “health perceived as normal”. However, comparison with the general Italian population [8] confirmed a reduction in the quality of life of MEN1 patients. These results are in accordance with the Berglund study [3], where Short Form-36 scores in General Health and Social Functioning were lower compared with population-based normative values. Our MEN1 sample reported lower scores in the 8 scales of SF-36, reaching significance (*p* < 0.05) only for limitations in social activities. The SF-36-measured quality of life in women is similar to that of men in most domains, resulting significantly lower (*p* < 0.05) only in physical activity. The oldest patients are those with the lowest SF-36 domain scores, with statistically significant differences (p < 0.05) in the level of physical activity, physical role, and physical pain, while the domains related to psychological and emotional health improve with increasing age, indicating that aging itself could represent a cause of worsening QoL.

In HADS-D, LOT-R and IES-R questionnaires, there are no significant differences in the average score for all the aspects analyzed, even when stratified by gender and age. In HADS-A, a statistically significant increase in anxiety (p < 0.05) with increasing age was found. There were no significant relationships between anxiety, depression, pessimism, intrusiveness, avoidance, and hyperarousal by gender, marital and working status of the patients, but educational level was associated with depression (*p* < 0.05). However, the heterogeneity of our MEN1 sample probably led to a low statistical comparison power, and it may have contributed to an underestimation of these differences.

An increase in the number of MEN1-related surgeries was associated with lower scores on the SF-36 questionnaire, with statistically significant differences (*p* < 0.05) in the level of physical role, body pain, general health, and social functioning (we did not evaluate the group with four surgeries because it was represented by only one patient). This confirms that multiple surgical interventions, which characterize the lives of almost all MEN1 patients, are one of the main conditions responsible for the worsening of QoL.

Results from the questionnaires were also associated with the number of drugs taken to treat MEN1 manifestations, to investigate if and how medical therapies could influence the QoL of patients. This analysis showed a significant increase in pessimism (*p* < 0.05) and a significant reduction in physical functioning (PF), role limitations due to physical health problems (RP), bodily pain (BP), general health perceptions (GH) and social functioning (SF) domains of the SF-36, associated with increasing numbers of medications. These results are different from those of Berglund [3], where the depression increased for patients with a high burden of disease and treatment.

Finally, our results showed a statistically significant increase in depression and in three sectors of SF-36 [in particular, vitality (VT), SF, and role limitations due to emotional problems (RE)] (*p* < 0.05), corresponding with increasing distances between the patients’ homes and the Referral Center, particularly between the distances of 0–200 km and 400–600 km. These data seem to confirm that even the perception of having easy access to constant, specialized, personalized care and follow-up significantly improves the psychological status and QoL of patients.

Comparison between MEN1 single and familial cases showed, in our cohort of patients, no significant differences in the result of the five questionnaires and in the distribution of anamnestic data (i.e. social status, clinical data, therapies, etc.). However, the absence of significant differences could be due to the relatively low number of single cases available for the study (only 9 on a total of 76 interviewed patients).

The inter-familial and intra-familial comparison of individual response to MEN1 diagnosis, showed mainly two different behaviors: 1) relatives of the index case who lived their life relatively well, thanks to the support of the family itself, the knowledge of what to expect and how to deal with the syndrome in the long term, and the fact to be aware of the possibility to be constantly followed-up in a dedicated Referral Center; 2) relatives of the index cases who lived their disease status with anxiety, stress and fear for the future, because they have the memory of the suffering of affected family members for multiple tumors and surgeries. Therefore, a personalized psychological support to MEN1-diagnosed patients should be considered within the medical plan for the most psychologically fragile individuals.

Unfortunately, specific questionnaires measuring HRQoL for MEN1 syndrome or other hereditary cancers have not yet been developed. Comparison of our results with previous studies was not easy, given that, in most cases, the studies used different questionnaires. HRQoL generic questionnaires are not developed for a complex tumor syndrome such as MEN1 and could lead researchers to underestimate or overestimate some specific traits of the disease that influence the psychophysical status of patients. The design of a MEN1-specific HRQoL questionnaire would help increase analytical effectiveness regarding this syndrome, and it would be strengthened by the collection of data on MEN1 patients from various countries.

## Conclusions

The main aim of this study was to estimate how the diagnosis and manifestations of a complex multi-tumor syndrome, like MEN1, could affect the quality of life of patients. Despite the presence of a rare pathology affecting patients throughout their lifetimes, results from the study indicated that our group of patients was moderately optimistic (50%), correlating with a QoL in the norm. We hypothesized that the relatively high percentage of “optimistic” approach could be due to the fact that, thanks to the dedicated Referral Center and the availability of personalized and constant follow-up, usually carried out by the same clinicians, patients feel involved in the decision-making process regarding their care, and consider their team well-informed on the management of their syndrome, giving them confidence that they will receive the best possible treatment.

Another factor that could explain the "positivity" of our study population, compared to those of previous studies in other countries, is the presence of an active national patient association, in which patients are able to share their clinical conditions and psychological perceptions, and express their fears and discomforts, without feeling ashamed and/or poorly understood, but, conversely, showing that "their scars" are a result of the paths their lives are taking, and that these things can happen, but they do not prevent the patients from living a normal life.

About 20% of patients cared for at the Referral Center refused to complete the questionnaires. No significant differences were reported, in terms of gender, age, disease presentation, surgeries and drug treatments, as well as marital status, education and employment status, between this group of patients and those who participated to the survey. It is possible that the people who did not participate in the study were, in most cases, psychologically "weaker" patients. Their “absence”, in the calculation of self-reporting HRQoL could be one of the reasons for our apparently “positive” results, meaning that the evaluated sample of patients was not representative of the entire population of our MEN1 patients. This fact could represent a bias in the study; therefore, the real emotional state of MEN1 patients could be lower than that obtained.

Moreover, the size of our MEN1 sample may have contributed to hiding statistical differences between different socio-demographic characteristics.

To resolve these issues and improve the statistical power of the analysis, the next step will be to extend the study nationally and internationally, providing an increased number of subjects to the sample analyzed. This will provide a more inclusive population and reduce underestimation of differences related to personal socio-demographic features.

Finally, the development of a dedicated MEN1 HRQoL questionnaire would be timely and possible.

## Methods

### Study design and population

This prospective monocentric and non-profit observational study was approved by the Investigation Board “Regional Ethical Committee for clinical trials in Tuscany—AREA VASTA CENTRO section” referral for the University of Florence (RIF 11213), and was fully endorsed by the Italian Society of MEN1 patients (Associazione Italiana Neoplasie Endocrine Multiple tipo 1 e 2). The study was carried out from January 2018 to January 2020.

The study population consisted of 76 Italian men and women with a clinical and/or genetic diagnosis of MEN1, referred and regularly followed-up at the Ambulatory of the Regional Referral Center for Hereditary Endocrine Tumors of the Tuscany Region, University Hospital of Careggi, Florence.

Clinical and genetic data for the 76 patients included in the study were retrospectively extrapolated from the MEN1 Florentine Patient Database, which includes data collected from 1991 to January 2020 [9].

All eligible patients (age ≥ 18 years) were invited to complete five questionnaires during a medical examination at the Center. Enrolled patients gave their informed consent for data collection and analysis; all data were made anonymous, and each patient was identified by a study-specific identification number. Data were strictly analyzed and published as aggregates. The impact of MEN1 and its diagnosis on the quality of life of these patients was measured through the administration of five self-compiled questionnaires. The questionnaires were administrated from January 2018 to January 2020.

### Socio-demographic questionnaire

This questionnaire aims to assess the impact of MEN1 on the patient's social sphere by gathering information regarding family status, education, social and working life.

### LOT-R

This is a test that measures dispositional optimism and pessimism, a shorter, but more objective and specialized, version of the LOT, consisting of only six questions. The brevity and objectivity of the LOT-R make it useful for cognitive and behavioral therapies. LOT-R has been successfully implemented to a broad range of populations, including adults fighting poverty, adolescents with depression, individuals suffering from social anxiety, and victims of trauma. It is by far one of the most accepted measures of optimism and positive thinking for adults and young people. The test has a strong internal consistency and it is characterized by a high degree of reliability [the Cronbach alpha value that measures this aspect is equal to 0.82 (minimum value 0.00 and maximum possible value 1.00)].

Respondents use a 5-point rating scale (0 = strongly disagree; 4 = strongly agree) to show how much they agree with 10 statements about positive and negative expectations. These statements include “In uncertain times, I usually expect the best” and “If something can go wrong for me, it will.” Four items are “filler” statements that are not scored.

Each response to the six questions of the LOT-R was associated, by clinicians, to a single score; the six single scores were then summed, thereby obtaining a total score ranging from 0 to 24 for each patient.

Interpretation of score was as follows: 1) 0–13 Low Optimism (High Pessimism); 2) 14–18 Moderate Optimism; and 3) 19–24 High Optimism (Low Pessimism).

The maximum score obtainable in the LOT-R is 24, which is an indicator of the patient being excessively optimistic and positive [10].

### IES-R

IES is a 15–item questionnaire that evaluates the impact of several traumatic experiences. The IES-R is a revised version of the IES, and was developed because the original version did not include a hyper-arousal subscale.

This survey consists of 22 questions aimed at assessing the emotional salience and trauma of an event. It is a self-assessment questionnaire that investigates the symptoms related to stress during the week preceding the evaluation. People facing stressful situations react with attitudes that oscillate between intrusiveness and avoidance. Avoidant behaviors are implemented on an unconscious level with the attempt to restore emotional balance, but often these attempts at defense are overwhelmed by intrusive thoughts and experiences. To restore stability, therefore, people tend to react by implementing hypervigilance strategies. Starting from this theory, the 22 items of the IES are divided into three subscales corresponding to the possible symptoms that characterize PTSD: intrusiveness, avoidance, and hypervigilance. Intrusiveness is assessed through questions that investigate the recurrence of the negative event in the form of images, perceptions and emotions and the recurrence of unpleasant dreams related to the event. Avoidance is investigated through items that evaluate how much the subject tends to avoid feelings, situations and ideas related to the event. Hyper-vigilance is assessed through questions that measure irritability or outbursts of anger, difficulty in maintaining concentration, excessive alarm responses, and difficulty falling asleep or maintaining sleep.

Responses have been singularly scored as: 0 = Not at all; 1 = A little bit; 2 = Moderately; 3 = Quite a bit; 4 = Extremely. Single response scores have been summed and evaluated as follows: 1) range 1–11: the patient presents little or no symptoms of post-traumatic stress. No action is required; 2) range 12–32: the patient presents several symptoms of post-traumatic stress. Patient monitoring is required; and 3) equal to or greater than 33: high probability of having a post-traumatic stress disorder; the patient should be referred for a more elaborate assessment [11].

### HADS

This is a self-reported rating scale of 14 items, designed specifically to measure anxiety and depression degree. Anxiety is defined as an unpleasant emotional state associated with the perception of a real threat and an unpleasant sense of imminent danger. Anxiety is a recognized common symptom following a diagnosis of cancer, which manifests with both physiological and psychological components. Depression can occur at any time in a patient with cancer, from the time of diagnosis and throughout treatment. The diagnosis of depression is based on mood, cognitive, physical, and behavioral symptoms. The mood symptoms are sadness, despair, and irritability. The cognitive symptoms are reduced ability to concentrate, memory problems, and negative thoughts (even suicide or extreme situations). Behavioral symptoms can be frequent crying, loss of appetite, sexual problems, and insomnia.

Seven items regard anxiety and seven regard depression. Total evaluation score is the sum of all 14 items (range 0–42). The score for each subscale (anxiety and depression) is the sum of the respective 7 items (range 0–21). Items referring to depressive symptoms that concern the somatic dimension of depression (e.g. insomnia, weight loss, fatigue) are excluded from the scale. Patients who obtain a score from 0 to 7 are indicated as normal; those with a score from 8 to 10 are indicated as patients at risk, which could turn into psychopathological cases (borderline), while patients with a score higher than 11 are indicated as major cases. The scale is only a means of screening for subsequent clinical examination [12, 13].

Patients are requested to read each sentence of the HADS and then indicate how often the situation described has occurred in the previous seven days. They express their evaluation by indicating a value ranging from 0 to 3. The patient is requested not to take too long to answer each statement, since the first, instinctive, answer is often considered to be the most accurate. Patients are instructed that there are no right or wrong answers and they should respond according to their feelings.

### SF-36

The SF-36 is a generic and multi-dimensional questionnaire aimed to investigate patient state of health and the HRQoL. It is characterized by its brevity (on average, the subject takes no more than 10 min to complete it) and accuracy (the instrument is valid and reproducible). SF-36 has discriminatory abilities against populations with psychiatric or physical problems and allows discrimination between groups of populations with severe medical conditions from groups of moderately ill or healthy populations.

The SF-36 questionnaire can be self-filled, but can also be the subject of a telephone or face-to-face interview. All but one of the SF-36 questions refers to a period of four weeks prior to completing the questionnaire.

SF-36 is articulated through 36 questions that allow you to assemble 8 different scales. The first three questions reflect physical health (PF—Physical functioning, RP—Role limitations due to physical health problems, BP—Bodily pain); the intermediate questions reflect general health (GH General health perceptions: VT—Vitality, energy or fatigue); the last 3 measure aspects of psychological and emotional health (SF—Social functioning, RE—Role limitations due to emotional problems, MH—General mental health, covering psychological distress and well-being). The calculated scores are then normalized on a scale of 0–100.

In five scales (PF, RP, BP, SF and RE), the state of health is described as the absence of limitations or disabilities; the maximum possible score of 100 is achieved when no limitation or disability is observed.

Three other scales (GH, VT and MH) are "bipolar", and measure a much wider range of health states, positive and negative. The intermediate score of 50 means that the subjects do not report any limitations or disabilities. A score of 100, on the other hand, is achieved only when the subjects report to have experienced positive health conditions and evaluate their health very favorably [14].

### Statistical analysis

Data from these questionnaires were collected in specific databases. Demographic and clinical characteristics of the study population were illustrated by using descriptive statistics, and data included in the database were expressed as percentages, averages, and relative standard deviations. Comparisons of continuous variables were statistically tested through the Independent T-test. A p value less than 0.05 was considered as an indicator of statistical significance for all the analyses performed.

## Data Availability

The datasets used and analyzed during the current study are available from the corresponding author on reasonable request.

## Abbreviations

BP: Bodily pain
FDO: Fear of disease occurrence
GEP-NETs: Gastro-entero-pancreatic neuroendrocrine tumors
HADS: Hospital Anxiety and Depression Scale
HRQoL: Health-Related Quality of Life
GH: General health perceptions
IES: Impact of Event Scale
IES-R: Impact of Event Scale-Revised
LOT-R: Life Orientation Test-Revised
LOT: Life Orientation Test
MEN1: Multiple Endocrine Neoplasia Type 1
MH: General mental health
PF: Physical functioning
PHPT: Primary hyperparathyroidism
PROMIS: Patient-Reported Outcomes Measurement Information System
PTSD: Post-traumatic stress disorder
QoL: Quality of life
RE: Role limitations due to emotional problems
RP: Role limitations due to physical health problems
SF: Social functioning
SF-36: Medical Outcomes Study 36-item Short-Form Health Survey
VT: Vitality
